# Molded Plywood with Proportions of Beech Bark in Adhesive Mixtures: Production on an Industrial Scale

**DOI:** 10.3390/polym16070966

**Published:** 2024-04-02

**Authors:** Roman Reh, Lubos Kristak, Jan Sedliacik, Pavlo Bekhta, Anita Wronka, Grzegorz Kowaluk

**Affiliations:** 1 Faculty of Wood Science and Technology, Technical University in Zvolen, 96001 Zvolen, Slovakia; kristak@tuzvo.sk (L.K.); sedliacik@tuzvo.sk (J.S.); bekhta@tuzvo.sk (P.B.); 2 Department of Wood-Based Composites, Cellulose and Paper, Ukrainian National Forestry University, 79057 Lviv, Ukraine; 3 Institute of Wood Sciences and Furniture, Warsaw University of Life Sciences, 02-776 Warsaw, Poland; anita_wronka@sggw.edu.pl (A.W.); grzegorz_kowaluk@sggw.edu.pl (G.K.)

**Keywords:** molded beech plywood, UF, ground beech bark, eco-friendly fillers, free formaldehyde emission

## Abstract

Molded plywood is used for furniture components such as seats, backrests, or integral seat shells, and it must be durable and harmless to health. Molded plywood is made with urea-formaldehyde (UF) adhesives; therefore, the issue of the fillers used in them is important. The potential of using ground beech (*Fagus sylvatica* L.) bark as an eco-friendly additive in UF adhesives for molded plywood manufacturing was investigated in this work. Wheat flour was used as a reference filler. The beech bark (BB) level as a filler was 10%, a value verified under laboratory conditions. Nine-layer flat and molded plywood were produced under industrial conditions from beech veneers bonded with a UF adhesive mixture. The mechanical (bending strength and bonding quality) and physical (swelling and absorbency values after 2 and 24 h) properties of the industrially fabricated molded plywood were evaluated and compared with the European standard requirements (EN 310 and EN 314-2). The mechanical properties of the molded plywood with the addition of BB in the adhesive mixture were acceptable and met these standards’ requirements. The positive effect of BB in the UF adhesive mixture on a reduction in formaldehyde emissions from the molded plywood was also confirmed. BB, considered to be wood-processing industry waste or a by-product, has significant potential to be used as a filler in UF resins for molded plywood production, providing an environmentally friendly, inexpensive solution for the industrial valorization of bark as a bio-based formaldehyde scavenger.

## 1. Introduction

Molded plywood is usually a three-dimensional product that is created from multiple beech (birch) veneer layers that are glued together using adhesive, heat, and pressure in a press. The veneer layers are arranged crosswise at an angle of 90°. Molded plywood is used for furniture components such as seats, backrests, or integral seat shells [1,2].

The main task of molded plywood is to maintain the shape in which it was made in the long term to properly serve its purpose. This is made possible by the fact that a grain-interlocking pattern of the adjacent veneers is applied, which greatly reduces swelling and shrinking and generates greater surface resistance [3,4].

The technological processes in the production of molded plywood are the same as in the manufacture of flat plywood; the difference is in the shape of the press plates used. In the manufacture of molded plywood, pressing molds, a matrix, and a patrix of the necessary shape are used and molded plywood is pressed between the surfaces. The condition is that both surfaces of the matrix and the patrix maintain the same distance when pressing when measured to the common perpendiculars, i.e., one surface must be equidistant from the other. Attention must be paid to the projection of the pressing pressure. The pressure in the surfaces perpendicular to its direction is manifested in full, but the pressure decreases with a deviation from the perpendicular direction about the angle forming the direction of the pressed surface. This is from a perpendicular direction to the direction of pressure [5,6]. These are facts of fundamental importance. This paper focuses on the composition of adhesive mixtures used to produce molded plywood, which must be suitable for the machinery and technological operations indicated. They must be such that they make a significant contribution to the long-term stabilization of the manufactured shape of the plywood and that they do not degrade over time [5,7].

In terms of applications of shaped pressing technologies of veneer materials, molded plywood is a product with a long history [5,8,9,10,11,12]. Therefore, the correct composition of the adhesive mixture used for molded plywood pressing is essential. Urea-formaldehyde (UF) adhesives are used for common shapes in molded plywood manufacturing because chairs made using them are used indoors [10,11,13,14,15,16,17].

UF adhesives create a sufficient bonding strength of veneers for molded plywood [9,15,18]. However, they are not used as one-component adhesives; there is always an adhesive mixture present in molded plywood production and, therefore, the resulting adhesive effect is also dependent on the proportion of other components of the UF adhesive mixture used [2,15,17].

Today’s modern UF adhesives are of significantly better quality than those produced and offered to molded plywood manufacturers in the 20th century. Formaldehyde emissions are notably already at relatively small levels, corresponding with strict technical standards.

The common method used to decrease free formaldehyde in the adhesive mixtures of molded plywood is the modification of the adhesive mixture using formaldehyde scavengers, whereby the scavengers are directly applied to the adhesive mixtures by mixing and by the related reaction [19,20,21,22].

The issue of minimum formaldehyde emission values from the adhesive mixtures used in molded plywood must be completely resolved before production in light of today’s strict technical standards because of the length of time pupils and students sit on chairs made from molded plywood during their daily school attendance. This is so constant that the health of pupils and students could be severely tested and endangered over the years [23,24,25,26]. Therefore, the use of scavengers in UF adhesive mixtures is of fundamental importance.

This paper describes research into the properties of molded (and flat) plywood produced in a factory. Both plywood types were pressed using disintegrated beech bark (BB) as a filler for UF mixtures. The industrial production of plywood was preceded by the thorough laboratory research testing of forest tree barks as fillers as well as their fractions and shares. This took place from 2013 to 2022 at the Technical University in Zvolen, Slovakia, mostly within its development workshops and laboratories. Only after rigorous laboratory tests and achieving optimal laboratory results was it decided to carry out the industrial tests in a full production operation. The industrial production of plywood using disintegrated beech bark as a filler for UF adhesive mixtures took place at the industrial plant BEKER-MP, s.r.o., Prešov, Slovakia (www.beker.sk, accessed on 23 February 2024).

Various flours are used in UF glue mixtures as fillers and they work well. The viscosity of such glue mixtures is reasonable, the industrial application of the adhesive mixtures is reasonably good, and the cost is not high [2,15,27]. Some scientists and researchers have attempted to replace flour with another type of filler that has all the effective properties of flour, but with the additional effect of effectively capturing the leakage of harmful formaldehyde from the adhesives. Some disintegrated bark of forest trees seems to fulfill this role [21,22,28,29].

Beech is a high-quality raw material used for plywood production and its bark is widely available in plywood mills because of debarking. Thus, this bark is present at the place of wood processing, but it is considered to be ineligible for further processing. The proposal to use it as a filler has given it a new purpose. Thus, BB was selected to produce molded plywood under industrial conditions (fully fledged production in a full industrial operation). BB has been proved to be an effective filler of adhesive mixtures to produce flat plywood under laboratory conditions and its chemical and technological parameters as well as collection, drying, and grinding methods have already been published [22,27,30,31,32,33].

## 2. Materials and Methods

### 2.1. Materials

Rotary cut beech (*Fagus sylvatica* L.) veneers with an average thickness of 1.30 ± 0.05 mm, 6% ± 1% moisture content (MC), and dimensions of 500 × 500 mm^2^ were used to produce the plywood. The raw wood material, which was used for peeling, came from the region of Jasov (Slovenské Rudohorie Mountains) in Slovakia. The veneers were made using a four-foot rotary lathe from KSB (Královopolská strojírna Brno, Czech Republic) at the industrial plant of DYHA TIROLA, s.r.o., Moldava and Bodvou, Slovakia (www.dyhatirola.sk, accessed on 23 February 2024).

As a binder, an industrial UF adhesive (Kronores CB 1100 F; DIAKOL Strážske s.r.o., Strážske, Slovakia) with about 67% dry content was used with an ammonium nitrate (NH_4_NO_3_) water solution (47%) as a hardener to reach the curing time to glue the mass at 100 °C in about 88 s [28]. The hardener was added at a ratio of 10 parts by weight (pbw) per 100 pbw of adhesive, according to common industrial formulations. The average adhesive viscosity to bond the veneers was 1220 mPa.s and its pH value was 8.6. Wheat flour (WF) was used as the reference filler (REF).

UF adhesive with 10 pbw per 100 pbw of WF based on a liquid UF resin was used as a reference sample. This concentration of fillers for molded plywood production was selected based on our long-term research; it achieved optimal values in the results of previous tests [21,22] and was consistent with the observations of other authors examining the issue of fillers in UF adhesive mixtures [27,29,34,35,36].

### 2.2. Methods

**Figure 1:**
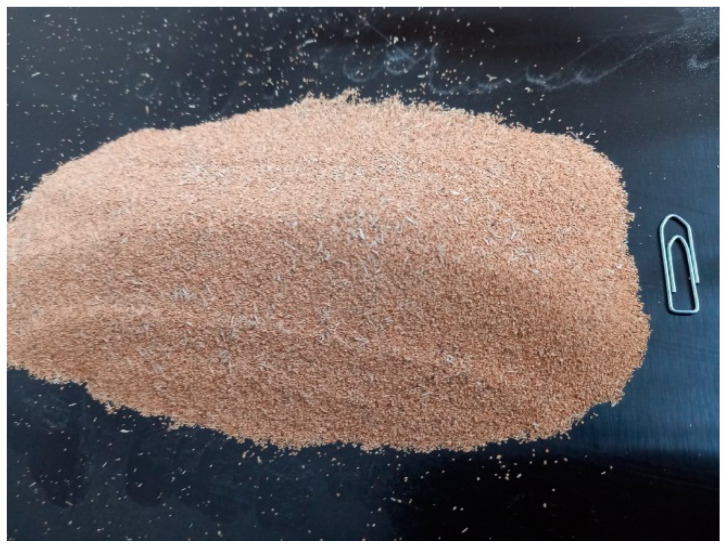
Beech bark in a dry state.

The beech bark was properly and finely ground (to a fraction of bark with grains smaller than 0.125 mm) (Figure 1). This was utilized as an alternative to WF; the same fraction of flour with grains smaller than 0.125 mm is used as a filler with a share of 10% in UF adhesives for plywood manufacturing. A higher bark content (15–20%) in adhesive mixtures has not been tested under industrial conditions because under laboratory conditions, it was found that the viscosity of such a mixture became problematic from the point of view of the perfect application of the adhesive mixture to the veneer surface.

Nine-layer flat (18 pieces) and molded (18 pieces) plywood samples were produced under industrial conditions with the use of the bonding mixtures listed in Table 1. The following parameters were applied during the plywood preparation: a bonding mixture spread of 150 g/m^2^, the adhesive mixture was then applied using a roller spreader, and a maximum press pressure of 1.4 MPa was used for the hydraulic press (Hydroma RM-3, Uherský Brod, Czech Republic). The pressing times are given in Table 1. The resulting thickness of all plywood after pressing was 10.0 ± 0.2 mm. All the produced flat and molded plywood samples were subjected to conditioning at 20 °C/65% ± 1% relative humidity (RH) to obtain a constant weight before further testing.

**Figure 2:**
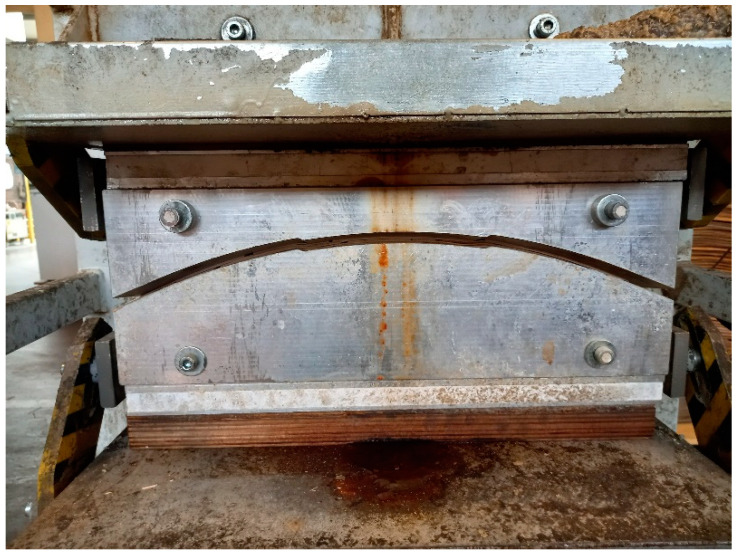
Molded plywood in the molded press during the pressing process.

Flat plywood was produced as a REF product on identical presses to the molded plywood. Their press plates were not molded; in this case, they were flat. The flatness of the plywood—and, therefore, their entire area—allowed us to produce a larger range of test samples than in the case of molded plywood production, where suitable places to produce test sample bodies were limited because of plywood roundness. Molded plywood was produced using molded press plates (Figure 2).

The following tests were completed for the produced flat plywood (REF): density (8 repetitions), bending strength (MOR) in a parallel direction to the grains of the face veneer layer (16 repetitions), MOR in a cross-direction to the grains of the face veneer layer (8 repetitions), bonding quality (8 repetitions), TS after 2 h (8 repetitions), TS after 24 h (8 repetitions), WA after 2 h (8 repetitions), and WA after 24 h (8 repetitions) [37,38]. We respected and observed the international conventions and procedures for the selection of samples from the panels. Every part of the panel had an equal chance of becoming a sample, but we omitted the edge of the board with a width of 50 mm and we performed this as objectively as possible [37,38].

The following tests were completed for the produced flat plywood (REF): density (8 repetitions), modulus of rupture (MOR) in a parallel direction to the grains of the face veneer layer (16 repetitions), MOR in a cross-direction to the grains of the face veneer layer (8 repetitions), bonding quality (8 repetitions), thickness swelling after 2 h (8 repetitions), thickness swelling after 24 h (8 repetitions), water absorption after 2 h (8 repetitions), water absorption after 24 h (8 repetitions), and free formaldehyde emissions [37,38].

The following tests were completed for the produced molded plywood: density (6 repetitions), MOR in a parallel direction to the grains of the face veneer layer (6 repetitions), MOR in a cross-direction to the grains of the face veneer layer (4 repetitions), bonding quality (6 repetitions), thickness swelling after 2 h (6 repetitions), thickness swelling after 24 h (6 repetitions), water absorption after 2 h (6 repetitions), water absorption after 24 h (6 repetitions), and free formaldehyde emissions [37,38]. The molded plywood properties were crucial in this research because we were studying the development of a new product using an alternative adhesive mixture; thus, the properties of the flat plywood produced using the same process and with the same adhesive mixture as the molded plywood served as the REF.

All mechanical tests were performed using a computer-controlled universal testing machine (TIRA 2200 Heckert Testing Machine, Schalkau, Germany) at the Technical University in Zvolen, Slovakia (within its own development workshops and laboratories). Physical tests were performed at the Technical University in Zvolen, Slovakia, as well. The free formaldehyde emissions were measured using the chamber method according to the standard EN 717-1 [39]. A UviLine SI 5000 spectrophotometer (SI Analytics, Plains, NY, USA) at 412 nm was used to determine the total formaldehyde content.

### 2.3. Statistical Analysis

An analysis of variance (ANOVA) and *t*-test calculations were used to test (α = 0.05) for significant differences between factors and levels, where appropriate. A comparison of the means was performed by employing the Duncan test when the ANOVA indicated a significant difference. The statistically significant differences for the achieved results are given in the Results and Discussion paragraphs, where the data are evaluated.

## 3. Results and Discussion

### 3.1. Density of Plywood Panels Produced under Industrial Conditions

As industrially produced plywood is usually produced in larger volumes and emphasis is placed on the production economics, some production operations can take place at a higher pace and less precisely than laboratory plywood production. Thus, in the first step, we produced the plywood under laboratory conditions with exact technological conditions and parameters and we then used these proven parameters in the production of the molded plywood in the factory. The achieved density results of the flat and molded plywood were reasonable and they are presented in Table 2.

### 3.2. Bending Strength of Plywood Panels

The results of the MOR tests are presented in Table 3.

The plywood MOR results were more than adequate in all cases. They were higher than the stated MOR values for standard plywood available on the market [40,41,42,43]. If we considered the type, corresponding plywood thicknesses, and number of veneer layers in the plywood, the achieved MOR values should have been at the level of 70–75 MPa in the case of the parallel direction to the grains of the face veneer layer and in the case of the cross-direction to the grains of the face veneer layer, at the level of 52–54 MPa. The achieved MOR values of the plywood industrially produced in the case of the parallel direction to the grains of the face veneer layer were higher by ±30% and in the case of the cross-direction to the grains of the face veneer layer, higher by ±15%. This applied to both the technical flour filler and ground BB filler. These were quite understandable values because plywood manufacturers tend to achieve higher values of mechanical strength of plywood than the minimum required. The values achieved in this research demonstrated that the tested adhesive mixtures had been properly developed and were suitable for the production of molded plywood. Statistically significant deviations did not occur for about 10% of filler content. Both tested fillers behaved in flat and molded plywood in much the same way in terms of MOR and were, therefore, suitable for the application. An increase in the filler content of about 5–10% significantly increased the viscosity of the binder; the mixture became problematic and possible problems with the even-spreading of the binder over the veneer surface could negatively influence the MOR of the plywood panels. Due to the results of previous laboratory research, we did not experiment with increasing the proportion of fillers in the adhesive mixtures in the plant. This avoided possible difficulties with applying adhesive mixtures or equipment damage.

### 3.3. Bonding Quality of Plywood Panels

The results of the bonding quality tests are presented in Table 4.

As in the case of MOR and also in the case of bonding quality, we compared the achieved values with those that were published as standards. Our results were more than adequate in all cases. They were higher than the stated bonding quality values for standard plywood available on the market [40,41,42,43] or in research papers [21,22,28,35,44,45]. If we considered the type, corresponding plywood thicknesses, and number of veneer layers in the plywood, the achieved bonding quality values should have been at the level of 0.8–1.4 MPa. The achieved bonding quality values of the plywood produced at the plant as part of this research were higher by ±65% in the case of a parallel direction to the grains of the face veneer layer. Both tested fillers behaved in much the same way in the flat and molded plywood in terms of the bonding quality and were, therefore, suitable for application. The fundamental differences in the break zones of the flat and molded plywood samples bonded with both types of filler pressed using the hot press process after the shear strength test did not occur. This could be explained by the fact that the proportion of filler of 10% in the adhesive was not large and, therefore, there was almost no difference between WF and disintegrated BB in the structure of the adhesive mixture used. This was also consistent with the observations of other authors who investigated the mechanical properties of plywood materials after modifications to the adhesive mixtures used to glue veneers or adhesive penetration in wood (veneers) [40,41,46,47,48,49,50,51,52,53,54,55].

### 3.4. Thickness Swelling and Water Absorption of Plywood Panels

The tested plywood was designed for indoor environments, yet the thickness swelling and water absorption values after 2 and 24 h were authoritative and informative.

The results of TS and WA of the flat and molded plywood panels after 2 and 24 h are presented in Table 5 and Table 6.

From Table 5 and Table 6 it is evident that TS and WA of both the flat and molded plywood after 2 and 24 h reached the standard values achieved by conventional plywood of this category and these thicknesses. The achieved values of WA and TS for the examined flat and molded plywood produced under industrial conditions did not significantly differ from each other, which corresponded with the idea that the industrially tested concentrations of adhesive mixtures and their composition were the results of long-term laboratory measurements and that there were no unexpected fluctuations in the physical properties tested [3,48,49]. Under the influence of water, the structure and properties of the adhesive mixtures in the cured state within the produced flat and molded plywood did not change. The extraction from the adhesive mixtures of water-soluble ingredients (mainly fillers, but also plasticizers, stabilizers, etc., in the adhesive) was at a reasonably suitable level [1,2,17,18,56,57,58].

### 3.5. Formaldehyde Emissions from Plywood Panels: Laboratory Tests at the Technical University in Zvolen

The results of the formaldehyde emission tests of plywood samples manufactured under industrial conditions are shown in Table 7. The obtained results clearly demonstrated that replacing wheat flour with beech bark as a UF adhesive filler led to a significant reduction in formaldehyde emissions. The reduction in formaldehyde emissions was approximately 24% compared with the control samples and approximately 66% compared with the requirements of the standard EN 636 [59]. It was encouraging that the use of both wheat flour and beech bark provided formaldehyde emissions for the E1 class of plywood as well as E0.5 class samples according to that standard, in alignment with the requirements of the German Chemikalien-Verbotsverordnung [60] for wood-based materials. The reduction in free formaldehyde emissions could be attributed to the presence of lignin and polyphenolic extractives in the chemical composition of tree bark [61]. Lignin can react with formaldehyde in an acidic medium [62], while polyphenolic extractives can react with formaldehyde even at an ambient temperature [63]. The potential of using tree bark to reduce the formaldehyde emissions from plywood panels has also been confirmed by other authors [21,22,28,31,44,46,63].

The indisputable advantage of using bark as a filler is that the bark is a residue from debarking logs. Moreover, the replacement of technical wheat flour in UF adhesive mixtures also allows for a wider use of food flour in the food industry, thus eliminating the a threat to the food security of the population.

### 3.6. Formaldehyde Emissions from Plywood Panels: Official Authorized Test in an Accredited Foreign Independent Laboratory

The official authorized test of formaldehyde emissions from molded plywood produced under industrial conditions using BB as a filler in the adhesive mixture took place in an accredited foreign independent laboratory. The results of the test were that molded plywood produced under industrial conditions using BB as a filler in the adhesive mixture achieved the following values:

1. According to EN 636: 2012 + A1: 2015, Class E1, the resulting value was 0.058 mg/m^3^ The limit is up to 0.124 mg/m^3^ [39,59].
2. According to the German Chemikalien-Verbotsverordnung standards for wood-based materials (enforcement dated 1 January 2020), the resulting value was 0.092 ppm. The limit is up to 0.1 ppm (the concentration of the EN 717-1 test was multiplied by a factor of 2) [60].

The fulfillment of the requirements according to both methods was satisfactory, with a large and clear margin from the limit value (decision rule: simple acceptance according to ILAC-G8: 09/2019, 4.2.1 [64]). The results of the test in an independent laboratory confirmed the laboratory tests of free formaldehyde emission measurements and the reliably low repeated measured values from the laboratories of the Technical University in Zvolen.

## 4. Conclusions

Beech bark (*Fagus sylvatica* L.) that is properly and finely ground (the fraction of bark with grains smaller than 0.125 mm) can be effectively utilized as an eco-friendly alternative to WF as a filler with a share of 10% in UF adhesives for plywood manufacturing. The determined values of the industrially produced flat and molded plywood panels with 10% bark content for MOR, bonding quality, and thickness swelling and water absorption after 2 and 24 h were equal to or higher than the mechanical and physical properties of commercially produced plywood and met the European standard requirements. A higher bark content (15–20%) in the adhesive mixtures was not tested under industrial conditions because under laboratory conditions, it was found that the viscosity of such a mixture became problematic from the point of view of the perfect application of the adhesive mixture to the veneer surface (due to high viscosity).

The positive effect of adding BB to the UF adhesive mixture on reducing harmful formaldehyde emissions in industrially produced plywood panels for two methods was also confirmed. After 669 h (four weeks), the detected value of free formaldehyde emissions from the tested molded plywood in an accredited foreign independent laboratory reached just under 47% of the permitted limit, according to EN 636: 2012 + A1: 2015. It also met the requirements of the German Chemikalien-Verbotsverordnung for wood-based materials by a sufficient margin. The results of the laboratory tests measuring emissions from molded plywood at the Technical University in Zvolen were similarly positive and the plywood clearly and reliably met the formaldehyde emissions requirements of E1 and, at the same time, met the formaldehyde emissions requirements of E0.5.

Based on the results presented, it can be concluded that BB, considered to be wood-processing industry waste or a by-product, has significant potential to be used as a filler in UF resins for molded plywood production, providing an environmentally friendly, inexpensive solution for the industrial valorization of bark as a bio-based formaldehyde scavenger. For academic researchers, the validation of the technology developed in industrial practice is encouraging, even more so when it comes to the successful development of a new product that is also ecological and renewable. It is sometimes difficult to find a willing partner in the industry to support operational testing. In this case, it succeeded, and many thanks go to the plant that is considering the permanent introduction of this technology and is currently developing a system of harvesting, drying, and grinding beech bark so that it is a process that is unpretentious and economically inexpensive. The assumptions and indications for this exist.

## Figures and Tables

**Table 1:** Compositions of adhesive mixtures, plywood molds, and selected pressing parameters.

Variant Label	Filler	Filler Content (pbw ^1^ per 100 pbw of Solid Resin)	Pressing Temperature (°C)	Pressing Time (min)
REF 10 flat plywood	Wheat flour	10	110	5
BB 10 flat plywood	Beech bark	10	110	5
REF 10 molded plywood	Wheat flour	10	110	5
BB 10 molded plywood	Beech bark	10	110	5

^1^ Pbw: parts by weight.

**Table 2:** The density of flat and molded plywood panels.

Variant Label	Filler	Filler Content (pbw ^1^ per 100 pbw of Solid Resin)	Densities (kg/m^3^)
REF 10 flat plywood ^1^	Wheat flour	10	758 (11) *
REF 10 flat plywood ^2^	Wheat flour	10	764 (6)
BB 10 flat plywood ^1^	Beech bark	10	751 (10)
BB 10 flat plywood ^2^	Beech bark	10	755 (6)
REF 10 molded plywood ^1^	Wheat flour	10	761 (21)
REF 10 molded plywood ^2^	Wheat flour	10	730 (22)
BB 10 molded plywood ^1^	Beech bark	10	754 (11)
BB 10 molded plywood ^1^	Beech bark	10	735 (5)

^1^ Parallel direction to the grains of the face veneer layer. ^2^ Cross-direction to the grains of the face veneer layer. * Standard deviations are in parentheses.

**Table 3:** Bending strength of flat and molded plywood panels.

Variant Label	Filler	Filler Content (pbw^1^ per 100 pbw of Solid Resin)	MOR (MPa)
REF 10 flat plywood ^1^	Wheat flour	10	95 (9) *
REF 10 flat plywood ^2^	Wheat flour	10	62 (3)
BB 10 flat plywood ^1^	Beech bark	10	93 (5)
BB 10 flat plywood ^2^	Beech bark	10	69 (3)
REF 10 molded plywood ^1^	Wheat flour	10	153 (22)
REF 10 molded plywood ^2^	Wheat flour	10	58 (2)
BB 10 molded plywood ^1^	Beech bark	10	93 (8)
BB 10 molded plywood ^2^	Beech bark	10	60 (4)

^1^ Parallel direction to the grains of the face veneer layer. ^2^ Cross-direction to the grains of the face veneer layer. * Standard deviations are in parentheses.

**Table 4:** Bonding quality of flat and molded plywood panels.

Variant Label	Filler	Filler Content (pbw ^1^ per 100 pbw of Solid Resin)	Bonding Quality (MPa)
REF 10 flat plywood ^1^	Wheat flour	10	2.5 (0.3) *
REF 10 flat plywood ^2^	Wheat flour	10	2.4 (0.1)
BB 10 flat plywood ^1^	Beech bark	10	2.8 (0.3)
BB 10 flat plywood ^2^	Beech bark	10	2.8 (0.3)
REF 10 molded plywood ^1^	Wheat flour	10	2.9 (0.0)
REF 10 molded plywood ^2^	Wheat flour	10	2.8 (1.5)
BB 10 molded plywood ^1^	Beech bark	10	3.1 (0.4)
BB 10 molded plywood ^1^	Beech bark	10	3.0 (0.2)

^1^ Parallel direction to the grains of the face veneer layer. ^2^ Cross-direction to the grains of the face veneer layer. * Standard deviations are in parentheses.

**Table 5:** Thickness swelling of flat and molded plywood panels after 2 and 24 h.

Variant Label	Filler	Filler Content (pbw ^1^ per 100 pbw of Solid Resin)	TS (%) (after 2/24 h)
REF 10 flat plywood ^1^	Wheat flour	10	1.4 (0.1)/3.3 (0.2) *
REF 10 flat plywood ^2^	Wheat flour	10	1.6 (0.1)/3.6 (0.1)
BB 10 flat plywood ^1^	Beech bark	10	1.6 (0.3)/3.6 (0.7)
BB 10 flat plywood ^2^	Beech bark	10	1.6 (0.1)/4.5 (0.3)
REF 10 molded plywood ^1^	Wheat flour	10	2.6 (0.9)/5.6 (1.0)
REF 10 molded plywood ^2^	Wheat flour	10	2.0 (0.1)/5.0 (1.0)
BB 10 molded plywood ^1^	Beech bark	10	2.3 (0.2)/4.7 (0.5)
BB 10 molded plywood ^1^	Beech bark	10	1.9 (0.1)/4.2 (0.3)

^1^ Parallel direction to the grains of the face veneer layer. ^2^ Cross-direction to the grains of the face veneer layer. * Standard deviations are in parentheses.

**Table 6:** Water absorption of flat and molded plywood panels after 2 and 24 h.

Variant Label	Filler	Filler Content (pbw ^1^ per 100 pbw of Solid Resin)	WA (%) (after 2/24 h)
REF 10 flat plywood ^1^	Wheat flour	10	14.7 (1.2)/33.0 (2.3) *
REF 10 flat plywood ^2^	Wheat flour	10	14.1 (1.0)/31.9 (1.5)
BB 10 flat plywood ^1^	Beech bark	10	12.8 (1.3)/28.8 (1.0)
BB 10 flat plywood ^2^	Beech bark	10	22.4 (1.3)/41.1 (0.4)
REF 10 molded plywood ^1^	Wheat flour	10	26.2 (2.6)/40.7 (2.0)
REF 10 molded plywood ^2^	Wheat flour	10	20.6 (1.0)/39.5 (2.1)
BB 10 molded plywood ^1^	Beech bark	10	15.6 (2.3)/37.8 (2.2)
BB 10 molded plywood ^1^	Beech bark	10	17.1 (2.8)/35.7 (3.6)

^1^ Parallel direction to the grains of the face veneer layer. ^2^ Cross-direction to the grains of the face veneer layer. * Standard deviations are in parentheses.

**Table 7:** Formaldehyde emissions of molded plywood panels.

Class of Formaldehyde Emission	Formaldehyde Emissions Using a UF Filler				Requirement According to EN 636 (mg/m^3^)	Requirement According to Chemikalien-Verbotsverordnung	
	Wheat Flour (mg/m^3^)	Beech Bark (mg/m^3^)	Wheat Flour (ppm)	Beech Bark (ppm)		(mg/m^3^)	(ppm)
E1	0.055	0.042	0.044	0.034	≤0.124	-	-
E0.5	0.055	0.042	0.044	0.034	-	0.062	0.050

## Data Availability

Data are contained within the article.
